# Comparing dose in the build‐up region between compensator‐ and MLC‐based IMRT

**DOI:** 10.1120/jacmp.v13i5.3748

**Published:** 2012-09-06

**Authors:** Khosrow Javedan, Geoffrey G. Zhang, Sarah Hoffe, Vladimir Feygelman, Kenneth Forster

**Affiliations:** ^1^ Radiation Oncology Moffitt Cancer Center, Tampa Florida Tampa, Florida; ^2^ Department of Chemical and Biomedical Engineering University of South Florida Tampa Florida

**Keywords:** build‐up dose, IMRT, Monte Carlo, compensator, MLC

## Abstract

The build‐up dose in the megavoltage photon beams can be a limiting factor in intensity‐modulated radiation therapy (IMRT) treatments. Excessive surface dose can cause patient discomfort and treatment interruptions, while underdosing may lead to tumor repopulation and local failure. Dose in the build‐up region was investigated for IMRT delivery with solid brass compensator technique (compensator‐based IMRT) and compared with that of multileaf collimator (MLC)‐based IMRT. A Varian Trilogy linear accelerator equipped with an MLC was used for beam delivery. A special solid brass step‐wise compensator was designed and built for testing purposes. Two step‐and‐shoot MLC fields were programmed to produce a similar modulated step‐wise dose profile. The MLC and compensator dose profiles were measured and adjusted to match at the isocenter depth of 10 cm. Build‐up dose in the 1–5 mm depth range was measured with an ultrathin window, fixed volume parallel plate ionization chamber. Monte Carlo simulations were used to model the brass compensator and step‐and‐shoot MLC fields. The measured and simulated profiles for the two IMRT techniques were matched at the isocenter depth of 10 cm. Different component contributions to the shallow dose, including the MLC scatter, were quantified. Mean spectral energies for the open and filtered beams were calculated. The compensator and MLC profiles at 10 cm depth were matched better than ±1.5%. The build‐up dose was up to 7% lower for compensator IMRT compared to MLC IMRT due to beam hardening in the brass. Low‐energy electrons contribute 22% and 15% dose at 1 mm depth for compensator and MLC modalities, respectively. Compensator‐based IMRT delivers less dose in the build‐up region than MLC‐based IMRT does, even though a compensator is closer to the skin than the MLC.

PACS number: 87.55.dk, 87.56.ng

## I. INTRODUCTION

Superficial dose, including the dose in the build‐up region for megavoltage beams, has been an area of interest in clinical radiation therapy since the inception of external beam radiotherapy.^(1)^ Among many treatment sites and techniques, the dose in the build‐up region is of interest for head and neck IMRT treatments.^(2)^ Excessive dose in the build‐up region can cause erythema and moist desquamation. An adverse skin reaction can lead to a treatment break which multiple studies have shown to be associated with worse local control due to tumor cell repopulation.^(3)^

Thus a significant clinical concern is avoiding excessive surface dose to potentially ensure better treatment compliance and outcomes. On the other hand, physicians are concerned that deliberately underdosing skin to avoid an adverse reaction may result in a local failure in some clinical settings.

The dose in the build‐up region is determined by the photon energy spectrum and the angular distribution of the photons and electrons. These parameters are not modeled well in commercial treatment planning systems (TPS). As a result, those TPS are known to be inaccurate in calculating dose in the build‐up region, as reported by Chung et al.^(4)^ These authors found that the two commercial TPS overestimated surface dose by 7.4% to 18.5%.

In the IMRT planning process,^(5)^ the ideal variable fluence maps are typically determined first. To convert this idealized fluence into a physically deliverable one, the beam can be modulated by either dividing it into a series of sub‐beams (segments) created by a multileaf collimator (MLC), or by inserting a variable‐thickness solid attenuator in the path of the beam (compensator‐based IMRT). Because of the uncertainty in the build‐up region calculations by the model‐based treatment planning systems, the differences in the superficial doses between the MLC‐based and compensator‐based IMRT cannot be accurately ascertained simply by comparing the treatment plans.

There are several contributing factors that can potentially cause the dose difference between the two techniques in the build‐up region. One is the scatter from the compensator. It has been previously shown^6^, ^7^ that the closer the compensator is to the patient, the larger is the scatter contribution to the build‐up region. On the other hand, the beam hardening by the compensator may decrease the superficial dose.

Measuring dose in the build‐up region poses unique challenges due to the lack of electronic (quasi)equilibrium. While parallel plate chambers are fairly well suited for dose build‐up measurements because of the minimal volume averaging effect, they are known to over‐respond at shallow depths.^(8)^ An extrapolation chamber has been known to provide better dosimetric results^(9)^ when compared to the parallel plate chambers, but it is bulky and time‐consuming to use. Velkey et al.^(10)^ and Rawlinson et al.^(11)^ have provided simple over‐response corrections for the parallel plate chambers.

Because of the challenges posed by measurements in the buildup region, Monte Carlo (MC) simulations are considered one of the more robust methods of determining the dose near the phantom surface.^(12)^ The objective of our work was to investigate the dose differences in the build‐up region between the MLC‐based and compensator‐based IMRT delivery techniques using a MC simulation program. The study identified and evaluated the contaminant radiation^13^, ^14^ (scattered photons and electrons) within the head of a linear accelerator for the two delivery techniques. The effect of this contaminant radiation on the dose in the build‐up region was investigated for varying compensator‐to‐surface distances (CSD), and the results were compared between the two techniques. Finally, energy spectra for the two delivery techniques were ascertained from the simulations.

## II. MATERIALS AND METHODS

### A. Compensator and MLC fields

**Figure 1:**
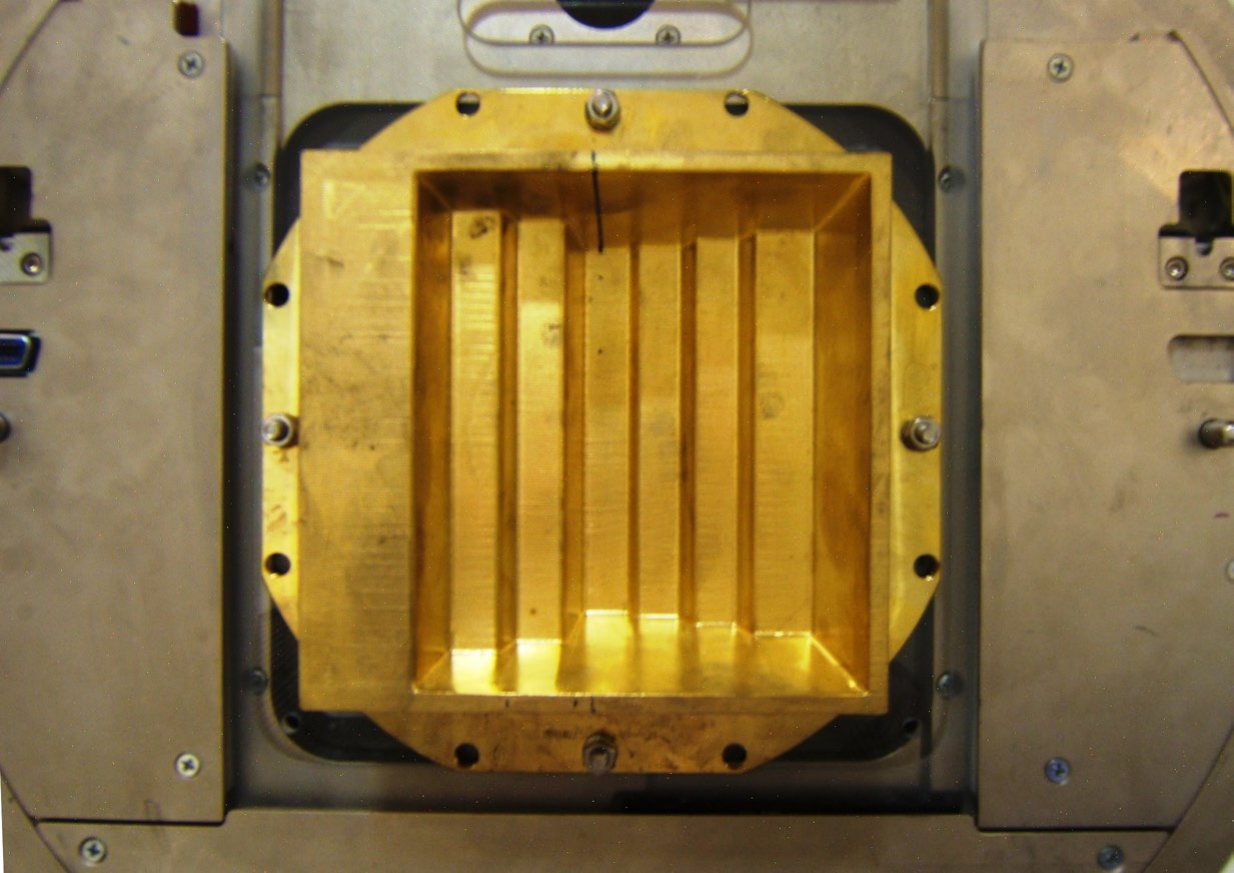
Brass compensator mounted on an open port Plexiglas tray inserted into upper wedge slot of an accelerator. From left to right side of the figure, the step thickness was 7.62, 5.08, 4.0, 0.6, 1.0, 2.0, and 3 cm. Step width varied from 2.5 to 3.4 cm projected at the isocenter. The effective jaw setting was $21.4\times 15.4\;{\text{cm}}^{2}$ at the isocenter.

A solid brass step compensator (Fig. 1) was fabricated by a commercial vendor (dotDecimal Inc., Sanford, FL) to deliver an intensity‐modulated step‐wise profile at 10 cm depth in water in a single field. The compensator was mounted on an open port Plexiglas tray and inserted into the accelerator accessory tray mount. A 6MV $21\times 15{\;\text{cm}}^{2}$ beam was delivered using a Trilogy linear accelerator (Varian Medical Systems, Palo Alto, CA). A Varian Millennium 120 leaf MLC was used to dynamically generate a similar step‐wise dose profile. The required MLC segments were created with Varian SHAPER program (v. 6.2). Due to the large field size exceeding the MLC leaf extension limits, the field had to be split in two to deliver the entire profile. To create a step‐wise profile using MLC technique that was similar to the one generated by the compensator‐based technique, a ratio of the physical beam attenuation of the brass compensator was used to relate the amount of beam‐on time per segment needed to create the profile. The dose index (fractional dose segment per total dose for a field) for each segment was estimated by using Eqs. (1) and (2).

(1) $$BS1_{i}=\frac{e^{‐\mu x1i}}{\sum_{i=1}^{N}e^{‐\mu x1i}}$$

(2) $$BS2_{i}=\frac{e^{‐\mu x2i_{i}}}{\sum_{i=1}^{N}e^{‐\mu x2i}}$$

where $BS1_{i}$ is the beam‐on time for segment *i* in the first MLC field, and $BS2_{i}$ is the beam‐on time for segment *i* in the second MLC field. For each MLC segment in the fields, the corresponding brass step thickness is *x1i* for segment *i* in the first field and *x2i* for segment *i* in the second field. The effective linear attenuation coefficient μ for brass was determined to be $0.375\;{\text{cm}}^{-1}$ by substituting the measured transmission ratio for a $10\times 10{\;\text{cm}}^{2}$ field into the exponential attenuation formula μ=‐ln(Transmission)/x, where *x* is the brass thickness in cm.

Equations (1) and (2) relate the fractional beam‐on time for the MLC segments to the physical thickness, and therefore attenuation, of the compensator steps. In order for the two MLC fields to produce the same step‐wise dose distribution as that of the compensator steps, we need to apply the segment weighting to each segment and add them together.

The segment weights SW1 and SW2 are calculated using Equations 3 and 4:

(3) $$SW1_{i}=BS1_{i}\frac{MU1}{MU1+MU2}$$

(4) $$SW2i=BS2_{i}\frac{MU2}{MU1+MU2}$$

where *MU1* and *MU2* are the monitor units for fields 1 and 2, respectively, which can be written as

(5) $$MU1=\frac{\sum_{i=1}^{N}e^{‐\mu x1i}}{\sum_{i=1}^{N}e^{‐\mu x1i_{i}}+\sum_{i=1}^{N}e^{‐\mu x2i}}$$

(6) $$MU2=\frac{\sum_{j=1}^{N}e^{‐\mu x2i}}{\sum_{i=1}^{N}e^{‐\mu x1i}+\sum_{i=1}^{N}e^{‐\mu x2i}}$$

The step‐wise IMRT profiles from the compensator‐based and the MLC techniques were measured at 10 cm depth using a commercially available linear diode array detector system, Profiler (Sun Nuclear Corporation, Melbourne, FL). The MLC‐based step‐wise profiles were matched to the compensator‐based IMRT profiles by adjusting the dose index for each segment and minor adjustments to the MLC positions.

After the relative profiles were matched, the final step was to obtain the same ionization charge on the central axis with an ionization chamber. An ultra‐thin window (0.02724 mm) fixed volume parallel plate ionization chamber (EXRADIN Model A10 Standard Imaging. Inc., Middleton, WI) was used. The chamber in the Plastic Water phantom (CIRS Inc., Norfolk VA) was positioned at the normalization point at isocenter at 10 cm depth (90 cm SSD). Based on the chamber readings, minor adjustment was made to the monitor units for the compensator delivery. After the absolute dose profiles were matched, chamber measurements were made in the build‐up region.

### B. Chamber measurements in the build‐up region

Chamber readings for each profile step were collected at 1, 3, and 5 mm depths in Plastic Water. The lateral shift for each step was made by using the lateral couch position readout at the accelerator console. The distance for each shift was double‐checked with a metal ruler. The chamber readings were normalized to the readings obtained during the profiles matching procedure described above. The chamber readings were corrected using the Rawlinson method^(11)^ to account for geometry and wall material density:

(7) $$P\prime (d)=P(d)=\frac{C(E)l}{W}\rho^{0.8}e^{\left(\frac{‐4d}{d_{\text{max}}}\right)}$$

where *P* ^/^ *(d)* is the corrected dose at depth *d, P(d)* is measured dose at *d*, the energy dependent factor *C(E)* is 0.27 for a 6 MV beam,^(11)^ *l* is the plate separation, *W* is the inner wall diameter, ρ is the wall material density, *d* is the depth to the front surface of chamber, and $d_{\text{max}}$ is the depth of maximum dose.

### C. Monte Carlo modeling

An EGSnrc‐based^(15)^ MC simulation package for clinical radiation treatment units, BEAMnrc^(16)^, was used.

#### C.1 Model validation

**Figure 2:**
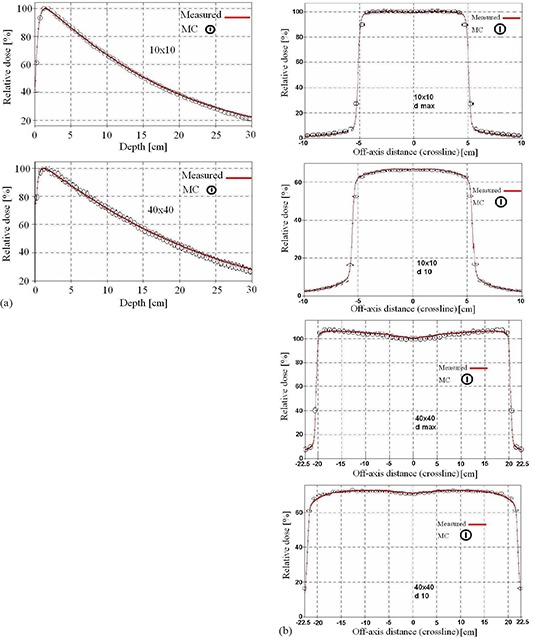
Comparison between measured and calculated percent depth dose curves and beam profiles for $10\times 10{\;\text{cm}}^{2}$ (a) and $40\times 40{\;\text{cm}}^{2}$ (b) fields. The profiles were compared at the depths of $d_{\text{max}}$ and 10 cm in water.

The percentage depth dose curves as well as the beam profiles in a water phantom from MC simulations were compared with the measured data for 5, 10, 20 and 40 cm open square field sizes. Representative examples of these comparisons (for a $10\times 10\;\text{and}\;a\;40\times 40\;{\text{cm}}^{2}$ field at $d_{\text{max}}$ and at a depth of 10 cm), are shown in Fig. 2.

After successful validation, phase space files for different jaw openings were generated, in which the physical parameters for all the particles traversing the plane of interest below the secondary jaws were stored. These files are then used as radiation sources for MLC and compensator simulations, obviating the need to resimulate the accelerator head each time.

#### C.2 Geometric modeling of the compensator and MLC fields

**Figure 3:**
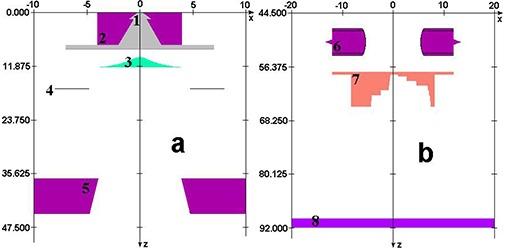
Monte Carlo model of a Varian Trilogy accelerator head geometry. Major parts such as the target (1), primary collimator (2), flattening filter (3), transmission chamber (4), and jaws (5) are shown in panel (a). MLC (6), the step wedge (7), and the phantom top (8) are shown in panel (b).

The component module BLOCK in BEAMnrc was used for the geometric modeling of the measured compensator physical dimensions, including divergence. The MLC was modeled using the appropriate component module in BEAMnrc. The accelerator models are shown in Fig. 3. The nominal widths of both the compensator steps, and MLC segments were slightly adjusted to match the simulated profiles to the measurement at 10 cm depth. Although small (a fraction of a millimeter), these adjustments were instrumental to obtaining a good match.

Phase space files calculated either below the MLC or the compensator were used as radiation sources for phantom dose distribution calculations using DOSXYZnrc.^(16)^ A flat water phantom was set up downstream from the phase space planes at either 32.4 cm CSD (90 cm SSD), or 48.4 cm CSD (106 cm SSD) to calculate the central axis depth doses and contributions of contaminant radiation for each MLC segment and the compensator. Appropriately weighted contributions from each MLC segment were added together to determine the cumulative modulated dose distribution under the MLC.

#### C.3 Segment weights

Step‐wise dose profiles were calculated using MC for both the MLC and brass compensator techniques. The exact weights of each MLC segment and the thickness of each brass step were again slightly adjusted as a part of matching with the measurements. The modifications were small, within the expected uncertainties of MC calculations. Finally, the calculated doses for the MLC‐ and compensator‐based modulation techniques were matched at 10 cm depth. The measured and calculated doses in the build‐up region and dose profiles at 10 cm depth were compared for the two delivery techniques.

#### C.4 Dose in the build‐up region as a function of compensator‐to‐surface distance (CSD)

MC simulations were done to approximate the build‐up dose difference with the CSD changes. This is only an approximation of the differences one would see with a different accelerator; as for an accurate estimation, a complete simulation of that accelerator would be required. To approximate the effects due to the CSD change, but not an SSD change, all the results were corrected using the Mayneord's F factor.^(17)^

#### C.5 Depth dose and contributions of contaminant radiation

The component module (CM), CHAMBER, was used as a phantom to facilitate the depth dose, as well as contaminant radiation dose calculations in BEAMnrc. Total dose and contaminant radiation doses were calculated in water at 1.0, 3.0, 5.0, 10.0, 15.0, 53, and 100 mm depths. The contaminant dose contribution was from scattered photons and electrons from the accelerator head and the MLC.

To investigate the effect of beam hardening by the compensator, the calculated energy fluence distributions were compared for a $2\times 15{\;\text{cm}}^{2}$ open field, and the same beam filtered by a 2 cm thick slab of brass.

## III. RESULTS

### A. Model validation

The Monte Carlo model was validated by matching the calculated and measured 6 MV beam profiles and percent depth dose curves for the small and large open fields at several depths in water. The percentage depth dose curves and profiles from MC simulations matched the measured data within ±1% for the low gradient dose region. In regions of build‐up or penumbra, the distance between the calculated and measured profiles was within 1 mm, with an example shown in Fig. 2.

### B. Step‐wise dose profile match at 10 cm depth

**Figure 4:**
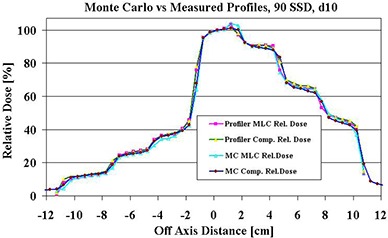
The matched step‐wise profiles produced by an MLC and a solid compensator. The center of 6 MV beam traverses the thinnest part (0.6 cm) of the brass compensator.

To collect the same ionization charge at the normalization point, a total of 729 MUs (600 MU for field 1+129 MU for field 2) were delivered with the MLC segments and 262 MUs with the compensator. Matched dose profiles at the isocenter (10 cm depth) are shown in Fig. 4. The disagreement between the measurements and calculated profiles did not exceed ±1.5%.

### C. Dose comparison in the build‐up region

The MC calculations and measured data agree within ±2% for compensator thicknesses of 1, 2, and 3 cm. The doses in the build‐up region at 1, 3, and 5 mm depths are presented in Tables 1(a), (b), and (c). The dose was consistently lower with the compensator‐based IMRT delivery compared to the MLC‐based delivery, with the maximum difference of 7% at 5 mm depth under the 2 cm thick brass compensator.

The p‐values for the paired t‐test between the calculated and measured doses for the compensator steps were 0.11, 0.18, and 0.63 for 1, 3, and 5 mm depths, respectively. The corresponding p‐values for the MLC steps were 0.27, 0.16, and 0.55. The difference between measured and calculated doses for compensator steps and MLC steps was not statistically significant (p>0.05).

The dose in the build‐up region was slightly lower for the compensator compared to the MLC technique under the 1.0 and 3.0 cm thick steps. The t‐test p‐values between the calculated doses for compensator and MLC steps were 0.009, 0.002, and 0.012 for 1.0, 3.0, and 5.0 mm depths, respectively. The corresponding values for the measured doses were 0.003, 0.001, and 0.009. The difference between the compensator and MLC doses corresponding to different steps was statistically significant (p<0.05).

**Table 1(a):** Doses (cGy) at 1 mm depth in Plastic Water and with the compensator and the MLC as a function of equivalent step thickness of the compensator. Doses were calculated with Monte Carlo and measured with parallel plate ionization chamber at 90 cm SSD, with the dose at isocenter being 100 cGy.

	*COMP*		*MLC*	
*STEP (cm)*	*Calculated*	*Measured*	*Calculated*	*Measured*
0.6	69.0±1.0	69.0±0.3	71.0±1.4	70.0±0.3
1.0	62.0±1.0	64.0±0.3	66.0±2.0	68.0±0.3
2.0	44.0±1.0	47.0±0.3	49.0±1.5	52.0±0.2
3.0	30.0±1.0	30.0±0.2	36.0±1.1	34.0±0.2
4.0	27.0±1.0	29.0±0.2	29.0±1.0	31.0±0.2
5.08	20.0±1.0	21.0±0.2	22.0±1.1	24.0±0.2
7.62	13.0±1.0	12.0±0.2	13.0±1.0	13.0±0.2

**Table 1(b):** Doses (cGy) at 3 mm depth in Plastic Water and with the compensator and the MLC as a function of equivalent step thickness of the compensator. Doses were calculated with Monte Carlo and measured with parallel plate ionization chamber at 90 cm SSD, with a dose at isocenter being 100 cGy.

	*COMP*		*MLC*	
*STEP (cm)*	*Calculated*	*Measured*	*Calculated*	*Measured*
0.6	113.0±1.7	109.0±0.3	116.0±2.3	113.0±0.3
1.0	100.0±2.0	98.0±0.2	104.0±2.1	102.0±0.3
2.0	70.0±1.8	71.0±0.3	76.0±2.3	75.0±0.2
3.0	50.0±1.5	48.0±0.2	55.0±1.7	53.0±0.2
4.0	39.0±1.2	39.0±0.2	41.0±1.2	43.0±0.2
5.08	27.0±1.1	27.0±0.2	31.0±1.2	31.0±0.2
7.62	15.0±1.0	15.0±0.2	16.0±1.0	15.0±0.2

**Table 1(c):** Doses (cGy) at 5 mm depth in Plastic Water with the compensator and the MLC as a function of equivalent step thickness of the compensator. Doses were calculated with Monte Carlo and measured with parallel plate ionization chamber at 90 cm SSD, with a dose at isocenter being 100 cGy.

	*COMP*		*MLC*	
*STEP (cm)*	*Calculated*	*Measured*	*Calculated*	*Measured*
0.6	136.0±2.0	133.0±0.2	138.0±2.8	135.0±0.2
1.0	118.0±1.8	119.0±0.2	123.0±2.8	122.0±0.3
2.0	82.0±1.6	83.0±0.2	89.0±2.3	90.0±0.2
3.0	58.0±1.5	57.0±0.2	62.0±1.9	60.0±0.3
4.0	45.0±1.1	46.0±0.2	47.0±1.6	49.0±0.2
5.08	32.0±1.1	32.0±0.2	34.0±1.4	35.0±0.2
7.62	17.0±1.0	16.0±0.2	17.0±1.0	16.0±0.2

### D. Contribution of contaminant radiation

Table 2 shows the contribution of contaminant radiation from the solid compensator and the step‐and‐shoot MLC calculated as a percentage of the total dose at selected depths at the central axis.

The photon dose contribution was 78%, 91%, 95%, and 99% of the total dose for the compensator and 82%, 92%, 95%, and 99% of the total dose for the MLC technique at 32.4 cm CSD and at 1.0, 3.0, 5.0 and 15.0 mm depths in water, respectively. At 48.4 cm CSD, the photon contribution was 82%, 93%, 96%, and 99% of the total dose for the compensator technique and 83%, 92%, 95%, and 100% of the total dose for the MLC, at the same set of depths.

The contaminant electron dose contribution was 22%, 9%, 5%, and 1% of the total dose for the compensator technique and 15%, 6%, 3%, and 0% of the total dose for the MLC delivery, at 32.4 cm CSD and at 1.0, 3.0, 5.0, and 15.0 mm depths in water, respectively. At 48.4 cm CSD, the contaminant electron dose contribution was 18%, 7%, 4%, and 1% of the total dose for the compensator and 15%, 6%, 4%, and 0% of the total dose for the MLC technique at the same depths.

MLC scatter dose contribution to the total dose was 3%, 2%, 2%, and 1% of the total dose at 32.4 cm CSD, at 1.0, 3.0, 5.0, and 15.0 mm depths in water, respectively. At 48.4 cm CSD, the MLC scatter dose contribution was 2%, 2%, 1%, and 1% of the total dose for the same depths.

**Table 4:** The total dose and percentage contribution from the photons, contaminant electrons, and MLC scatter are shown as a function of depth for the two IMRT techniques at (a) 32.4 cm CSD (90 cm SSD) and (b) 48.4 cm CSD (106 cm SSD).

*IMRT*	*Depth (cm)*	*Total Dose cGy*	*Photons %*	*Contaminant Electrons %*	*MLC Component %*
Solid Compensator (a)	0.1	69.0±2.5	78	22	0
	0.3	113.0±2.0	91	9	0
	0.5	136.0±1.8	95	5	0
	1.0	155.0±1.7	98	2	0
	1.5	160.0±1.6	99	1	0
	5.3	132.0±1.0	100	0	0
	10.0	100.0±1.0	100	0	0
Step‐and‐shoot MLC (a)	0.1	71.0±2.8	82	15	3
	0.3	116.0±2.7	92	6	2
	0.5	138.0±2.5	95	3	2
	1.0	152.0±2.2	98	1	1
	1.5	157.0±2.2	99	0	1
	5.3	130.0±2.0	99	0	1
	10.0	100.0±1.0	99	0	1
Solid Compensator (b)	0.1	50.0±2.5	82	18	0
	0.3	79.0±2.2	93	7	0
	0.5	92.0±1.9	96	4	0
	1.0	108.0±1.8	98	2	0
	1.5	110.0±1.8	99	1	0
	5.3	93.0±1.0	100	0	0
	10.0	70.0±1.0	100	0	0
Step‐and‐shoot MLC (b)	0.1	54.0±2.9	83	15	2
	0.3	81.0±2.7	92	6	2
	0.5	96.0±2.4	95	4	1
	1.0	108.0±.20	98	1	1
	1.5	111.0±2.0	99	0	1
	5.3	93.0±1.0	100	0	0
	10.0±1.0	72.0±1.0	100	0	0

**Figure 5:**
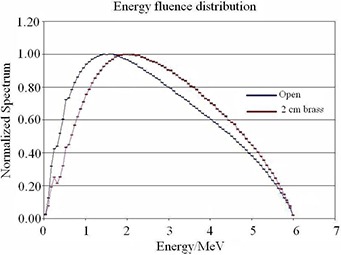
Normalized planar energy fluence distribution of 6 MV beam for $2\times 15{\;\text{cm}}^{2}$ field with and without a 2 cm brass slab.

The photon beam spectra for an open beam and a beam attenuated with a 2 cm thick brass compensator are shown in Fig. 5. The average energy increased from 1.57±0.10 MeV for the open beam to 2.17±0.10 MeV for the beam filtered by the 2 cm of brass compensator.

## IV. DISCUSSION

This study was limited to tests conducted with a simple step‐wise brass compensator and a corresponding MLC segment arrangement producing a similar fluence profile. This corresponds to fairly simple IMRT fields, and the results should not be automatically extrapolated to the IMRT beams with higher degree of modulation, particularly when a significant number of MLC segments are less than 2 cm in width.

The sources of the dosimetric differences at shallow depths between the MLC and compensator modulators can be only quantified with MC calculations, where the contributions of the different particles can be evaluated separately. However, Monte Carlo simulations also have limitations. As the dose calculation grid needs to become finer to accurately calculate the steep dose gradients in the build‐up region, the number of particles interacting in these thin slabs diminishes. Under these circumstances, to perform a calculation with small uncertainty a prohibitively large number of histories would be required to achieve the requisite statistics. The input file for the individual MLC segment simulations in this work was restarted 7 times to achieve better statistics.

The simulation showed that the dose in the build‐up region was lower under the 2 cm thick compensator compared to an MLC segment arrangement providing the same degree of modulation. This dose reduction was due to beam hardening produced by the compensator, as evidenced by the photon spectra comparison (Fig. 5).

**Figure 6:**
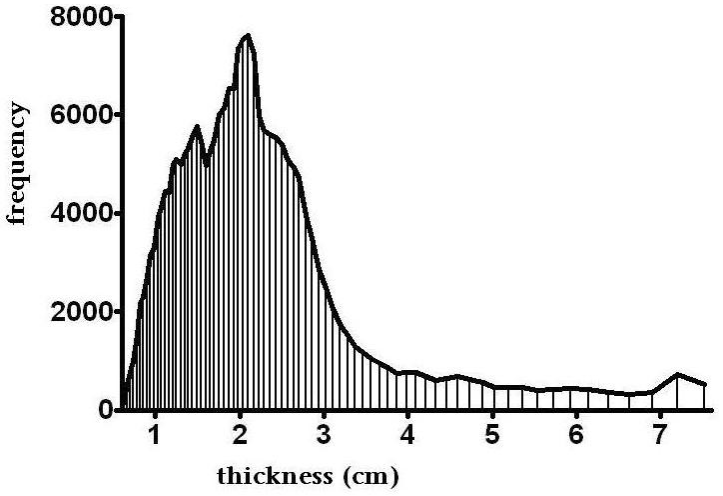
Compensator thickness analysis based on 50 retrospective IMRT fields (Opp et al.(18), with permission).

In support of having selected the 2 cm thick compensator for dose comparisons, in a separate but related study, Opp et al.^(18)^ have analyzed previously planned cases using IMRT with brass compensators. In that work, a histogram of transmission factors (plotted by compensator thickness) for 10 cases (with a total of 50 brass compensators) was generated. It is clear that the most probable compensator thickness in the modulation region was about 2 cm, as shown in Fig. 6.

The closer the compensator is to the patient, the higher skin dose the patient would get, due to the scattered low energy photons and electrons. In all accelerator configurations, the brass compensator is always closer to the patient than the MLC is. However, MC simulations in this study indicate that for the modulation level equivalent to 2 cm of brass, the compensator dose was 5%, 6%, and 7% lower than the MLC dose at 1, 3, and 5 mm depths, respectively. Extended SSD measurements and simulations in this study suggest that the use of accelerators other than Varian, which can afford larger compensator to surface distance, would further reduce the dose in the build‐up region when using compensators.

## V. CONCLUSIONS

Low‐energy scattered photons and electrons are the major contributors to dose in the build‐up region near the surface. The dose from contaminant electrons sharply decreases in magnitude with depth, while the photon contribution increases with depth in the build‐up region. This trend was similar for both 32.4 and 48.4 cm CSDs (90 and 106 cm SSDs), except the dose in the build‐up region was reduced at extended CSD, particularly with the compensator. The beam hardening effect in compensator‐based IMRT reduces the number of low‐energy photons in the treatment beam which, in turn, reduces the shallow dose. Even with a smaller distance to patient skin compared to that of MLC‐based IMRT, compensator‐based IMRT still delivers lower build‐up dose, which could be beneficial for skin sparing in certain radiotherapy treatments.
